# Author's reply

**DOI:** 10.4103/1319-3767.45070

**Published:** 2009-01

**Authors:** M. P. Desarda

**Affiliations:** Hospital and Research Centre, 18, Vishwalaxmi Housing Society, Kothrud, Pune 411 029, India. E-mail: desarda@hotmail.com

Sir,

I am thankful to Naguib, *et al*.[1] for reading my article[2] with interest. I have gone through their comments carefully. However, it appears that the authors misunderstand the concept between the tension present at rest and the tension present during contraction of any normal muscle. As is well understood, the muscles of the human body are relaxed at rest and the tension (increased tone) is created only during contractions. The strip of the external oblique aponeurosis in our operation is also relaxed (without tension) at rest and the tension (increased tone) is created only during contractions. This is a normal physiological phenomenon. Therefore, our surgical technique is a tension-free inguinal hernia repair. This does not occur in the other described techniques of the pure tissue hernia repairs since the muscles are pulled down from their normal location to suture to the inguinal ligament. This creates tension on the suture line even at rest and, moreover, is aggravated in multiples during the contractions of those muscles.

Additionally, in my study, it is clearly stated that there was no recurrence or groin pain following the surgery. Therefore, it is misleading on the part of Naguib, *et al*. to state that we made a claim of a low recurrence rate. In a recent article dealing with a series of 860 patients having 920 inguinal hernias[2] with a follow-up period of more than 7 years, I have demonstrated that these results compared well with other international publications. Continuous non absorbable sutures were used in all my previous surgeries.

The thin fascia covering the external oblique aponeurosis helps to keep the fibers together, if at all there is any chance of separation. This basic property of the fascia is generally known to the readers.

Finally, with regard to the claim by the authors that the repair depends on the aponeurotic sheath of the external oblique as the only posterior layer and that the original weak posterior wall has been left repaired is incorrect. They also state that suturing the edge of the upper flap to the posterior wall does not strengthen the posterior wall muscles. However, the posterior wall is not formed by only transversalis fascia, as is generally described, but it is composed of two layers. Transversalis fascia forms the posterior layer and is papery thin without any strength except at some places where it is thickened to form the iliopubic tract. The second layer is in front and that is formed by the aponeurotic extensions from the transversus abdominis aponeurotic arch.[3,4] There is no muscle in the posterior wall, as claimed by the authors, therefore, the question of strengthening the muscle does not arise. The sutured strip in the new technique replaces the absent or deficient aponeurotic element to form the new posterior wall. As such, their comment that the posterior wall is not repaired is incorrect. The statement by the author Naguib *et al*. that muscles run in different directions even in the inguinal canal region is also inaccurate and should be supported by evidence. The new concepts of the physiology of the inguinal canal based on surgical anatomy has been previously demonstrated in a series of 200 patients undergoing hernial repair surgery.[5]

Finally, issues like definition of discomfort was meant heavy feeling without pain and risk of inguinal floor dissection was meant trauma to iliac vessels or ilio-pubic vein or any aberrant vessel causing troublesome bleeding. Observations pertaining to “learning curve” or “interrupted sutures” in other no-mesh techniques are unwarranted since the article in question does not aim to compare this technique with another.

## References

[1] Naguib (2008). No-mesh Inguinal Hernia Repair with Continuous Absorbable Sutures: Is it a Step Forward or Backward?. Saudi J Gastroenterol.

[2] Desarda MP (2008). No-mesh inguinal hernia repair with continuous absorbable sutures: A dream or reality? A study of 229 patients. Saudi J Gastroenterol.

[3] Desarda MP (2006). Physiological repair of inguinal hernia: A new technique (Study of 860 patients). Hernia.

[4] Anson BJ, McVay CB (1971). Surgical anatomy.

[5] Anson BJ, Morgan EH, McVay CB (1960). Surgical anatomy of the inguinal region based upon a study of 500 body-halves. Surg Gynaecol Obstet.

[6] Desarda MP (2003). Surgical physiology of inguinal hernia repair: A study of 200 cases. BMC Surg.

